# Risk factors for alcohol use among adolescents: The context of township high schools in Tshwane, South Africa

**DOI:** 10.3389/fpubh.2022.969053

**Published:** 2022-10-19

**Authors:** Bonolo Mmereki, Mmampedi Mathibe, Lindiwe Cele, Perpetua Modjadji

**Affiliations:** ^1^Department of Public Health, School of Health Care Sciences, Sefako Makgatho Health Sciences University, Ga-Rankuwa, South Africa; ^2^Non Communicable Diseases Research Unit, South African Medical Research Council, Cape Town, South Africa

**Keywords:** alcohol use, risk factors, adolescents, township high schools, Tshwane, South Africa

## Abstract

**Background:**

Risk factors for alcohol use originate from many interconnected factors to an interplay of social and physical environments. However, there is a scarcity of data on the contextual risk factors of alcohol use among the adolescents regarding high schools located in townships in South Africa. This study aimed to determine the risk factors for alcohol use among adolescents attending selected township high schools in Tshwane, South Africa.

**Method:**

A validated researcher-administered questionnaire was used to collect data on the demographics, as well as current alcohol use, types, quantity, and frequency among adolescents (*n* = 403) in the three high schools. Data were analyzed using STATA 17.

**Results:**

The response rate was 97%, with the mean age of 16 ± 2 years for the adolescents. Forty-eight % (48%) of the adolescents reported current alcohol use, which was associated with sex, age, number of children, school grade, repeated grade, spare time job and types, having a pocket money to school, child social grant, transport mode to school, and smoking. The odds of current alcohol use were higher for adolescents in grade 10 [AOR = 6.71; 95% CI: 3.16–14.24], grade 11 [AOR = 4.45; 95% CI: 2.21], grade 12 [AOR = 3.05; 95% CI: 1.47–6.31], repeating a grade [AOR =2 .20; 95% CI: 1.32–3.67), and working during a spare time [AOR = 2.91; 95% CI: 1.33–6.37]. Both sexes had higher odds of alcohol use in the ages of 15–17 and 18–21 years, than adolescents aged 13–14 years.

**Conclusion:**

Key risk factors for alcohol use among learners were sex, age, school grade, repeated grade, and working during a spare time. More evidence-based interventions that would have a greater impact in addressing alcohol use among adolescents, such as focusing on availability, marketing, and taxation of alcoholic beverages, are necessary.

## Introduction

Globally, alcohol remains the drug of choice among adolescents, mostly accompanied by other substance abuse (1, 2). The World Health Organization's Global Burden of Disease has reported alcohol and illicit drug use as the main risk factors for cause-specific disability-adjusted life years for young people aged 10–24 years (3). Hence, underage drinking has become a pressing public health issue leading to destructive behaviors that threaten their lives and others (1, 4–6). Almost half of the school-age adolescents (49.6%) in South Africa have at least consumed alcoholic beverage in their lifetime (5, 7). The prevalence of alcohol use among school adolescents in the country ranges from 22 to 53.8% (8, 9). Binge drinking is a rapid and excessive drinking over a short period of time (10) and has been estimated at 15–32% (11–14). Notwithstanding that 12% of the young generation has reported alcohol use before the age of 13 years, despite the South African National Liquor Act prohibiting alcohol sale to individuals younger than 18 years of age (15).

Substantial discrepancies in the prevalence of alcohol use among school adolescents in the 9 provinces (Eastern Cape, Free State, Gauteng, KwaZulu-Natal, Limpopo, Mpumalanga, Northern Cape, North West, and Western Cape), different settings, and racial groups in South Africa have been reported, which suggests that there are variances in the contributing factors of alcohol use, such as socio-cultural and demographic factors (4, 13, 16–18). Some of the socio-demographic factors implicated on alcohol use include age, race, smoking, missing school because of illness, self-reported academic/education, repeated school class/grade, difficulty use of leisure time, a lack of healthy recreational activities, low socioeconomic status, and attending religious services (13, 16, 19–21). However, as most of these earlier studies were conducted in urban and rural settings, the extent to which these findings are relevant for adolescents from townships (i.e., peri-urban) communities is limited. The lifestyle behavior of alcohol use in adolescents is a result of the influences of risk factors, which may be personal factors, suggesting that continuous scientific research to study them is imperative (22).

Adolescents are prone to making changes to the extent that they feel socially accepted (23). As a result, their emotional instability by unfamiliarity and fear of new experiences or decision-making and low self-esteem can lead them to alcohol and illicit drug use, school problems, unprotected sex, legal problems, emotional changes, traffic accidents, suicides, and homicides (24). Furthermore, the risk of damage to organs, such as the brain, liver, and kidneys, and sexual dysfunction due to alcohol consumption is higher among the adolescents than in adults (25). Literature documents that young people who drink alcohol before the age of 15 years are more likely to perform poor at schools, drop out, develop alcohol dependence, as well as experiencing mental and social harm, and cardiovascular diseases, later in adulthood (26).

Most concerning is that high schools are believed to create some social and cultural surroundings, which may encourage alcohol use among the adolescents (4, 16, 27, 28). Nonetheless, schools are also known to offer an opportune setting health education and health promotion interventions (29). Worth noting is that adolescence is a suitable age for interventions that can enforce delayed alcohol use and avert the beginning of social and health consequences that are social and health-related during adulthood (7, 30). The importance of healthy lifestyle behaviors to cardiovascular health promotion, risk reduction, as well as disease prevention and management is well established. Health behaviors, including alcohol and tobacco use, in addition to other behaviors, such as patterns of dietary intake, physical activity, and inactivity, have been universally emphasized and embraced as a central component of evidence-based guidelines for various population groups, including adolescents (31).

However, there is a dearth of recent data on the risk factors for alcohol use among adolescents in the South African township setting, except for minimal research (16, 18, 27), compared to several studies conducted in other settings (4, 8, 12, 13). School environments contribute significantly to adolescent alcohol, tobacco, and other substance disorders. A growing body of literature focuses on school connectedness and school climate as substance use determinants (32, 33). This study addressed the phenomenon of alcohol use among adolescents by addressing this research question: What are the contextual risk factors for alcohol use among adolescents studying in township high schools in Tshwane, South Africa, considering that setting has influence on lifestyle? Therefore, this study aimed to determine the risk factors for alcohol use among adolescents attending selected township high schools in Tshwane, South Africa.

## Methods and materials

### Study design and framework

A cross-sectional study anchored on the Socio-Ecological Model (34) as a basis for understanding the risk factors for alcohol use was conducted among the adolescents. This model posits that environmental influences on behavior fall into four broad domains: micro-system, meso-system, exo-system, and macro-system, and interactions within and between these domains determine behavior. For the purpose of this article, we focused on the exo-system, which includes the larger contexts within which the individual operates, such as the neighborhood organization (34, 35). Hence, high schools were considered relevant to access the adolescents.

### Study setting and population

The study was conducted in Soshanguve and Winterveld townships, located in the City of Tshwane municipality, in Gauteng Province. In a South African context, townships are under-developed residential areas that were reserved for mostly Africans to live during apartheid (36). Townships were designed as dormitory towns for the labor required to serve the needs of mining and other industries and had limited social services and even less economic infrastructure. Whether long-established or newly developed, formal or informal, most of these housing settlements still have several challenges in common, including the lack of social and economic facilities required to build sustainable communities, the continued distances, geographically and socioeconomically, from South Africa's economic heartlands (37). The residents in the townships where the study was conducted reside in both formal and informal settlements, with low socioeconomic status (4).

Schools in Soshanguve and Winterveld townships are under the Tshwane Department of Education, which is divided into four districts; Tshwane North, West, and South, and Gauteng North. Soshanguve township, located in Tshwane North, has 16 high schools, while Winterveld township in Tshwane West has six high schools, making a total of 22 high schools. These high schools are situated within 45 km in the North of Pretoria, the Capital City of South Africa (i.e., Pretoria), and are managed by the National Department of Basic Education. Each of the schools has roughly 800–1,100 learners' enrollment number and offers academic and recreational programs. Schools in Soshanguve and Winterveld townships belong to quintile three (Q3), which receive the majority of their funding from the South African government (38).

### Study participants

We adopted the adolescents' age categories from Kids First Pediatric Partners (39), roughly divided into three stages: early adolescence, generally ages (11–14 years); middle adolescence, ages (15–17 years); and late adolescence, ages (18–21 years). Therefore, learners who were attending high schools in Soshanguve and Winterveld townships at the time of the study were considered, from 13 years and above, with a written parental consent to participate in the study, and gave assent. The study excluded learners who could not obtain parental written consent.

### Sample size and sampling techniques

The study used a validated Raosoft^®^ online sample size calculator (Raosoft Inc., Seattle, WA, USA), which takes into consideration the Cochran formula for sample size determination. Sample size estimation was based on a population size of ±24 000 learners in the 22 high schools in Tshwane North and Tshwane West, 5% margin of error, 95% confidence level, and 50% response distribution. A minimum recommended sample for the study was 379, and to cater for a non-response, the sample was buffered with 10% and increased to 416.

A multistage sampling was used to select the high schools and the learners: First, randomly selecting three high schools out of the 22 schools, followed by a random selection of learners from the lists of learners who have obtained parental consent. First, the high schools were stratified by the size of enrollment, and three largest schools were selected. Each selected school was treated as a unit of analysis with a sample size of not < 120 learners to avoid disproportionate sampling among the three selected schools. Taking note of parental consent, we grouped participants by their grades and the participating students were randomly selected from each grade. Participants were randomly selected from the lists of learners per grade, and class, who have obtained parental consent in the three high schools. To be noted, the daily running of the three high schools was not interrupted during recruitment and data collection.

### Study instrument

A validated researcher-administered questionnaire used in the study to collect data was adapted from the previous studies conducted on substance abuse (4, 16, 40). The questionnaire was validated through content and face validity, through the use of the two experts in the field of substance abuse. Independent translators who speak Setswana, as their mother tongue and are conversant with English, did forward and backward translations of the questionnaire. Reliability of the instrument was ensured through pilot study in one of the high schools, conducted among 30 adolescents, which is documented as a suitable sample size of a pilot study (41). The results of the pilot study were not included in the data analysis for the main study. Prior to the pilot study, the research assistants who speak Setswana were taken through the process of conducting preliminary interviews in a local language, for training purposes by the main researcher. After pretesting the questionnaire, there were no changes to the content except for minimal clarity of wording, and simplifying layout and style. The questionnaire was comprised of the demographic information of adolescents, such as sex, age, school grade, repeated school grade, mode of transport to school, spare time, and recreational activities, as well as child support grant and religion. Further information was collected on alcohol use, types, quantity, and frequency, as well as tobacco use. Information was collected by the research assistants based on activities in the past 1 month. Research assistants interviewed learners one by one, while they were sitting in their respective classrooms/grades with desks far apart, after school. Four hundred and six adolescents responded out of 416 learners recruited, making a 97% response rate. For data analysis, three questionnaires with missing data above 10% were excluded, and the final sample size of 403 was used in the study.

### Procedures

Recruitment of the learners was done through school visits. During the visit, the researcher provided the principals of schools with letters addressed to the school governing bodies, ethical certificate from Sefako Makgatho Health Sciences University Research and Ethics Committee (SMUREC), permission letter from Tshwane Department of Education to access Pretoria North and West high schools, information leaflet, and the research proposal. Following the approval by the school governing bodies, the researcher liaised with the nutrition teacher in each selected school, for the process of obtaining parental consent for learners, as well as selecting learners from classes, and engaging them on the procedure of the study and preparations for data collection.

### Data entry and analysis

STATA 17 (StataCorp. 2015. Stata Statistical Software:Release 17. College Station, TX, USA) was used to analyze data. Complete case analysis was used to identify participants with missing data during analysis. Descriptive statistics in the form of frequencies (n) and percentages (%) were used to describe the sample and to determine the prevalence of current alcohol use and its components. Chi-square (χ^2^) was used to test for differences in proportions by gender and current alcohol use. Hierarchical logistic regression analysis was used to assess the associations of current alcohol use with demographic factors. The purposeful selection process began with a univariate analysis of each variable. Any variable having a significant univariate test at some arbitrary level was selected as a candidate for the multivariate analysis. We base this on the Wald test from logistic regression and a *p*-value cut-off point of 0.20 (42), suggested as an appropriate level. Researchers have reported that more traditional levels, such as 0.05, can fail in identifying variables known to be important (43, 44). A stepwise backward elimination process was used, and the iterative process of variable selection entailed removing non-significant from the model. At the end of this iterative process of deleting, refitting, and verifying, the model contained significant covariates (45). The results are presented as adjusted odds ratios (95% confidence interval) (AOR (95%CI). Probability was set at *p* < 0.05.

### Ethical considerations

This study was conducted according to the guidelines laid down in the Declaration of Helsinki (46), and all procedures involving human subjects were approved by Sefako Makgatho Health Sciences University Research and Ethics Committee (SMUREC/H/51/2018: PG). Furthermore, this study received permission from Tshwane Department of Education (Ref 8/4/4/1/2). Written informed consent was obtained from parents of all adolescents, whether younger than 18 years or older, and assent was obtained from the younger adolescents, while those aged 18 years gave written consent, prior to data collection.

## Results

### Characteristics of the adolescents

Four hundred and three adolescents participated in the study, and female adolescents [*n* = 218 (54%)] were more than male adolescents [*n* = 185 (46%)]. The mean age of adolescents was 16 ± 2 years. Adolescents were further divided into three age groups 13–14 years, 15–17 years, and 18–21 years (39). Table 1 shows the demographic characteristics of the adolescents and comparison by sex using a chi-square test. Most of the male adolescents were significantly older (aged 15–17 years and 18–21 years), compared to female adolescents, who were aged between 13 and 14 years (*p* = 0.001). A significant numbers of female adolescents were in Grade 8, compared male adolescents, while most male adolescents were grades 10 and 12 compared to female adolescents (*p* = 0.048). Male adolescents have significantly repeated a grade compared to female adolescents (*p* = 0.001). Female adolescents were doing most of housekeeping, compared to male adolescents who were significantly working at car wash and doing garden during a spare time (*p* = 0.004). Income/week for a job in a spare time was higher (>R150) among male adolescents (*p* = 0.016), who were more into recreational activities (*p* = 0.003) and sports (*p* = 0.001), compared to the female adolescents. More male adolescents had a weekly pocket money of more than R100 (*p* = 0.018) and walked to school (*p* = 0.006), as a mode of transport, as compared to female adolescents.

**Table 1:** Comparison of demographics of the adolescents by sex.

**Variables**	**All (*n* = 403) *n* (%)**	**Female (*n* = 218) *n* (%)**	**Males (*n* = 185) *n* (%)**	**χ^2^**	***P*-value**
Age (years)					
13–14	100 (25)	67 (30)	33 (18)	15.18	0.001*
15–17	169 (42)	95 (44)	74 (40)		
18–21	134 (33)	56 (26)	78 (42)		
School grade					
8	92 (23)	61 (28)	31 (17)	9.6	0.048*
9	90 (22)	49 (23)	41 (22)		
10	57 (14)	25 (11)	32 (17)		
11	91 (23)	49 (23)	42 (23)		
12	73 (18)	34 (16)	39 (21)		
Repeated grade					
No	272 (67)	168 (77)	104 (56)	19.83	0.001*
Yes	131 (33)	50 (23)	81 (44)		
Dwelling place					
Brick house	284 (70)	147 (67)	136 (73)	1.58	0.454
RDP house	72 (18)	43 (20)	29 (16)		
Shack	47 (12)	27 (12)	20 (11)		
Spare time job					
No	365 (91)	203 (93)	162 (88)	3.61	0.057
Yes	38 (9)	15 (17)	23 (12)		
Types of job in spare time					
Housekeeping	9 (24)	8 (53)	1 (4)	15.27	0.004”*
Car Wash	5 (13)	0 (0.0)	5 (22)		
Selling	8 (21)	3 (20)	5 (22)		
Gardening	4 (10)	0 (0.0)	4 (18)		
Other	12 (32)	4 (27)	8 (34)		
Income/week for a job					
in spare time					
R0	15 (39)	8 (53)	7 (30)	8.56	0.016*
R1–R150	5 (13)	4 (27)	1 (4)		
>R150	18 (47)	3 (20)	15 (65)		
Recreation activities					
No	183 (45)	114 (52)	69 (37)	9.08	0.003*
Yes	220 (55)	104 (48)	116 (63)		
Type of recreation activity					
None	183 (45)	114 (52)	69 (37)	26.31	0.001*
Sports	145 (34)	54 (28)	91 (49)		
Youth groups	75 (17)	50 (23)	25 (14)		
Transport mode to school					
Parents' car	6 (2)	6 (3)	0 (0.0)	11.98	0.006”*
Bus	71 (18)	46 (21)	25 (14)		
Taxi	77 (19)	45 (20)	32 (17)		
Walk	249 (61)	121 (56)	128 (69)		
Receiving pocket money/week					
R0	68 (17)	29 (13)	39 (21)	8.03	0.018*
R1–R100	281 (70)	165 (76)	116 (63)		
>R100	54 (13)	24 (11)	30 (16)		
Number of children					
0	381 (94)	205 (94)	176 (95)	1.16	0.560”
1	19 (5)	12 (5)	7 (4)		
2	3 (1)	1 (0.5)	2 (1)		
Receiving child social grant					
No	384 (95)	207 (95)	177 (96)	0.12	0.733
Yes	19 (5)	11 (5)	8 (4)		
Religion					
Non-Christian	305 (76)	45 (21)	53 (29)	3.49	0.062
Christian	68 (24)	173 (79)	132 (71)		

n stands for number of participants, % stands for percentage, RDP stands for Reconstruction and Development Programme, *stands for *p*-value: significant at 0.05, and ” indicates Fisher's exact test used for variables with expected values < 5 in a cell.

Table 2 shows the comparison of current alcohol and tobacco use among adolescents by sex. Types of alcoholic beverages reported were beer/cider, wine, spirits, and traditional beer, of which beer/cider [*n* = 106 (26%)] was the commonly used type. The prevalence of current alcohol use among the adolescents was 48%, and more male adolescents were alcohol users (56%) compared to female adolescents (41%) (*p* = 0.004). Smoking was estimated at 18% and more male adolescents were smokers (26%) as compared to female adolescents (8%) (*p* = 0.001), while cannabis was the mostly used smoking products than cigarette (*p* = 0.029).

**Table 2:** Comparison of current alcohol and tobacco use and products among adolescents by sex.

**Variables**	**All (*n* = 403) *n* (%)**	**Females (*n* = 218) *n* (%)**	**Males (*n* = 185) *n* (%)**	**χ^2^**	***P*-value**
Current alcohol use					
No	210 (52)	128 (59)	82 (44)	8.31	0.004*
Yes	193 (48)	90 (41)	103 (56)		
Types of alcoholic beverages					
Beer/cider	106 (55)	49 (54)	57 (55)	0.29	0.962
Wine	53 (27)	26 (29)	27 (26)		
Spirits	19 (10)	8 (9)	11 (11)		
Traditional beer	15 (8)	7 (8)	8 (8)		
Estimated quantity of alcohol use					
1–2 glasses/week					
3–4 glasses/week	65 (34)	36 (40)	29 (28)	3.02	0.221
Binge drinking	38 (20)	16 (18)	22 (21)		
	90 (47)	38 (42)	52 (51)		
Frequency of alcohol use					
Occasional drinkers	146 (76)	72 (80)	74 (72)	3.62	0.195”
Weekend drinkers	44 (23)	18 (20)	26 (25)		
Everyday drinkers	3 (2)	0 (0.0)	3 (3)		
Smoking					
No	160 (82)	83 (92)	77 (74)	11.04	0.001*
Yes	34 (18)	7 (8)	27 (26)		
Smoking products					
Cigarette	13 (38)	0 (0.0)	13 (48)	5.46	0.029”*
Cannabis	21 (62)	7 (100)	14 (52)		

n stands for number of participants, % stands for percentage, *stands for *p*-value: significant at 0.05, and ” indicates Fisher's exact test used for variables with expected values < 5 in a cell.

### Factors associated with current alcohol use

Older learners (*p* = 0.001), learners in grade 11 (*p* = 0.001), and who repeated grade (*p* = 0.001) used alcohol compared to their counterparts. More alcohol users had a spare time job (*p* = 0.020) and worked mostly at car wash and selling (*p* = 0.040). Alcohol users were more of those who walked to school (*p* = 0.001), received pocket money >R100 (*p* = 0.001), received child social grant (*p* = 0.032), had more than one child (*p* = 0.038), and smoking (*p* = 0.030) compared to the non-users (Table 3).

**Table 3:** Comparison of characteristics of adolescents by current alcohol use.

**Variables**	**Alcohol non users (*n* = 210) *n* (%)**	**Alcohol users (*n* = 193) *n* (%)**	**χ^2^**	***P*-value**
Sex				
Females	128 (59)	90 (41)	8.31	0.004*
Males	82 (44)	103 (56)		
Age (years)				
13–14	78 (37)	22 (12)	45.32	0.001*
15–17	87 (42)	82 (43)		
18–21	45 (21)	89 (46)		
School grade				
8	68 (32)	24 (12)	63.6	0.001*
9	66 (31)	24 (12)		
10	17 (8)	40 (21)		
11	29 (14)	62 (32)		
12	30 (14)	43 (22)		
Repeated grade				
No	167 (80)	105 (54)	28.92	0.001*
Yes	43 (20)	88 (46)		
Dwelling place				
Brick house	147 (71)	137 (71)	1.22	0.544
RDP house	31 (16)	31 (16)		
Shack	25 (13)	25 (13)		
Spare time job				
No	194 (94)	168 (87)	5.39	0.020*
Yes	13 (6)	25 (13)		
Types of job in spare time				
Housekeeping	6 (46)	3 (12)	9.27	0.040*
Car Wash	1 (8)	4 (16)		
Selling	0	8 (32)		
Gardening	2 (15)	2 (8)		
Other	4 (31)	8 (32)		
Income/week for a job				
in spare time				
R0	4 (31)	11 (44)	5.36	0.108
R1–R150	4 (31)	1 (4)		
>R150	5 (38)	13 (52)		
Recreation activities				
No	96 (46)	87 (45)	0.02	0.898
Yes	114 (54)	106 (55)		
Type of recreation activity				
None	96 (46)	87 (45)	2.16	0.34
Sports	70 (33)	75 (39)		
Youth groups	44 (21)	26 (13)		
Transport mode to school				
Parents' car	5 (2)	21 (11)	15.92	0.001*
Bus	50 (24)	36 (19)		
Taxi	41 (20)	1 (1)		
walk	114 (54)	135 (69)		
Receiving pocket money/week				
R0	27 (13)	41 (21)	13.02	0.001*
R1–R100	163 (78)	118 (61)		
>R100	20 (10)	34 (18)		
Number of children				
0	204 (97)	177 (92)	5.8	0.038”*
1	5 (2)	14 (7)		
2	1 (1)	2 (1)		
Receiving child social grant				
No	205 (98)	179 (93)	5.32	0.032*
Yes	5 (2)	14 (7)		
Religion				
Non-Christian	44 (21)	54 (28)	2.7	0.105
Christian	166 (79)	139 (72)		
Smoking				
No	0	160 (83)	4.73	0.030”*
Yes	1 (100)	33 (17)		

n stands for number of participants, % stands for percentage, RDP stands for Reconstruction and Development Programme, *stands for *p*-value: significant at 0.05, and ” indicates Fisher's exact test used for variables with expected values < 5 in a cell.

In the unadjusted model at a *p*-value cutoff point of < 0.20, sex, age, school grade, repeating a school grade, having a job in a spare time, mode of transport to school, receiving child social grants, and religion were associated with current alcohol use. A multivariate model was built using the covariates (i.e., sex, age, school grade, repeating a school grade, having a job in a spare time, mode of transport to school, child social grants, and religion). The results showed that the odds of alcohol use were 6.71 higher for adolescents in grade 10 [AOR = 6.71; 95% CI: 3016–14.24], 4.45 time higher for those in grade 11 [AOR = 4.45; 95% CI: 2.21], and 3.05 higher for those in grade 12 [AOR = 3.05; 95% CI: 1.47–6.31] than those in grade 8. Repeating a grade predisposed adolescents to alcohol use twice [AOR = 2.20; 95% CI: 1.32–3.67), as much as those who did not repeat a grade. The odds of alcohol use were 2.91 times higher for adolescents who had a job during their spare time [AOR = 2.91; 95% CI: 1.33–6.37]. Female adolescents aged 15–16 years [AOR = 3.55; 95% CI: 1.78–8.31], 18–21 years [AOR = 8.37; 95% CI: 3.56–19.65], and having a job during their spare time [AOR = 4.08; 95% CI: 1.24–13.44] were more likely to use alcohol than adolescents aged 13–14 years and not having a job during spare times, respectively. Male adolescents aged 15–16 years [AOR = 2.84; 95% CI: 1.14–7.04] and 18–21 years [AOR = 4.59; 95% CI: 1.68–12.50] were more likely to use alcohol than adolescents aged 13–14 years (Table 4).

**Table 4:** Association of alcohol use with covariates among the adolescents.

	**Crude OR (95% CI)**	***P*-value**	**Adjusted OR (95% CI)**	***P*-value**
ALL				
School grade				
8	1 (Reference)			
9	1.03 (0.53–1.99)	0.929	0.89 (0.45–1.77)	0.748
10	6.67 (3.20–13.89)	0.001*	6.71 (3.16–14.24)	0.001*
11	6.06 (3.19–11.50)	0.001*	4.45 (2.21–8.94)	0.001*
12	4.06 (2.10–7.85)	0.001*	3.05 (1.47–6.31)	0.003*
Repeating a grade				
No	1 (Reference)			
Yes	2.47 (1.35–4.50)	0.003*	2.20 (1.32–3.67)	0.003*
Job during a spare time				
No	1 (Reference)			
Yes	3.25 (2.10–5.05)	0.001*	2.91 (1.33–6.37)	0.007*
FEMALES				
Age (years)				
13–14	1 (Reference)			
15–17	3.29 (1.59–6.82)	0.001*	3.55 (1.78–8.31)	0.001*
18–21	6.92 (3.07–15.59)	0.001*	8.37 (3.56–19.65)	0.001*
Job during a spare time				
No				
Yes	2.26 (0.77–6.58)	0.136	4.08 (1.24–13.44)	0.021*
MALES				
Age (years)				
13–14	1 (Reference)			
15–17	3.14 (1.29–7.66)	0.012*	2.48 (1.14–7.04)	0.024*
18–21	6.00 (2.43–14.82)	0.001*	4.59 (1.68–12.50)	0.003*
Repeating a grade				
No	1 (Reference)			
Yes	2.47 (1.35–4.50)	0.003*	1.52 (0.75–3.07)	0.242

## Discussion

This study gave insight into the risk factors for alcohol use among adolescents attending selected township high schools in Tshwane, South Africa. The key findings were poor demographic status among the adolescents, high prevalence of alcohol use, binging, and beer/cider being the most used alcoholic beverage, as well as cannabis smoking. Further results on the risk factors for alcohol use among adolescents in the township high schools were sex, age, school grade, repeated grade, and working during a spare time. It is well documented that school-age adolescents are faced with alcohol-related harms associated with social, health, and educational problems (10). The high prevalence rates of alcohol use reported in this study are higher than the reported prevalence in several South African studies (4, 13, 27), yet lower compared to the findings in other studies (12, 14). However, almost similar estimates on the prevalence of alcohol use have been reported among school-aged adolescents in South Africa (16). Mohale and Mokwena (16) conducted a quantitative cross-sectional survey among learners in the four high schools of a peri-urban suburb south of Johannesburg and substances were more prevalent in male adolescents at 52%, and alcohol use was 51% among female adolescents. While the pooled prevalence of alcohol use among the adolescents in sub-Saharan countries (SSA) has been estimated at 32.8% (47), in European countries the prevalence has been estimated at 90% among school adolescents in their lifetime (48). This study further showed the gender disparity on alcohol use among the male and female adolescents and that adolescents aged 15–17 years and 18–21 years were using alcohol more than those aged 13–14 years.

Significant gender and age differences reported in other studies conducted among adolescents in South Africa, that male and older adolescents, and in higher school grades are more likely to use alcohol and to engage in binge drinking than female and younger adolescents and in low school grades (1, 11, 12, 49). As adolescents grow, they begin to make their own decisions and sometimes engage in unhealthy behaviors, such as drinking alcohol (50). The concern is that early drinking onset is linked to unintentional injuries, motor vehicle crashes, physical fights, unplanned and unprotected sex, nicotine dependence, illicit substance use, antisocial personality, conduct disorder, and academic underachievement (51–53). Further concerns pertain to the negative impact of alcohol use on the schooling progress of the adolescents, including the behavior of using alcohol later in adulthood (24, 26, 54).

In addition, this present study showed a high prevalence of binge drinking, similar to other studies in South Africa (15–32%) and attributed to several factors, such as the socioeconomic and environmental factors, as well as alcohol availability and accessibility (12, 13, 55, 56). In particular, the South Africa Youth Risk Behavior Survey (14) has reported almost similar prevalence for binge drinking to the current study higher among male adolescents than female adolescents. Binge drinking predisposes to diverse acute health harms, such as alcohol poisoning, alcohol-related blackouts and injury, alcohol-related physical and sexual assault, increased risk for sexually transmitted infection, and problems at school. Simultaneous use of other substances (e.g., cannabis) has been attributed to binge drinking (57–59). Given the relatively high numbers of binge drinkers, it would be useful to repeat this study looking at adolescents who engage in binge drinking to see associated risk factors among this higher risk group.

Adolescents in this study commonly consumed beer/cider and wine. The different types of alcoholic beverages reported in the current study are consistent with several studies in South Africa and SSA countries. In SSA, including South Africa, beer and wine, with addition to the spirit, are mostly consumed (60). Similarly, the reported types of alcoholic beverages in the current study are consistent to the findings of other local studies (16, 27, 28, 61, 62), and in African countries, such as Ghana (63) and Ethiopia (64). Easy access to alcohol, cigarette, and cannabis has been reported in South Africa (65), Nigeria (66), and India (66), including alcohol being a sign of maturity among adolescents (67). Cannabis, use in particular, has been implicated on increased risk for impairments in neurocognitive functioning, which may lead to negative consequences in school (e.g., trouble retaining information), impaired driving, and risky decision-making (68).

The risk factors for alcohol use reported in this study were similar to some studies conducted in different settings in South Africa (4, 13, 27, 69) and other countries (19, 70) based on academic achievement associated with alcohol use. In the present study, drinking was higher in grade 10 than in grades 11 and 12, and this could be due to the fact that adolescents in grade 10 are aged around 13–14 years, an average age range for starting to consume alcohol in South Africa. Data from the South African Youth Risk Behavior Survey found that, nationally, 25.1% (23.3–27.0) of Grade 8–11 learners had drunk five or more drinks of alcohol within a few hours on one or more days in the month preceding the survey (14). With regard to adolescents working during spare time as a risk factor for alcohol use, there is a consensus in the empirical literature that teenage employment, particularly, results in harmful consequences, such as lower school grades and diminished educational ambitions, among others. There is even more consensus that work puts adolescents at great risk of committing delinquent acts and other problem behaviors, such as smoking cigarettes, drinking alcohol, and using marijuana and other drugs (71).

Through a number of different pathways, alcohol use can influence educational outcomes (19, 72–75). These include the three following mediating factors: biological factors, behavioral factors, and emotional or mental health factors. First, alcohol use and related diseases (such as mental health issues) may have a direct biological effect on cognitive function and concentration at school. Alcohol has been shown to cause neurodegeneration and impaired functional brain activity (19) and can create learning and recognition problems (76). Second, alcohol use can lead to behaviors that affect educational performance, such as lower attendance or commitment. For example, alcohol use has been shown to be associated with absenteeism from school (74), less time spent on studying, and lower school attendance (75). Third, emotional or mental health factors related to alcohol use can affect educational performance. Alcohol use has been shown to negatively affect relationships with other students and teachers and commitment to school work (73). For instance, alcohol use by students may increase the odds of disengaging from school (such as through truancy or school suspension), which may in turn favor connections with antisocial peers. However, the relationship between alcohol use and educational outcomes is complex and multidirectional and has inverse relationship, where students who do less well in school may be more likely to engage in binge drinking as a coping mechanism (72).

Adolescents have until recently been overlooked in global health and social policy, one reason why they have had fewer health gains with economic development than other age groups (77). The possibility of this generation of adolescents being the healthiest depends on better childhood health, nutrition, and lifestyle, among others (31, 77). Alcohol use during the rapid transitions and growth of adolescence has implications on health (78). Early initiation of alcohol use might lead to alcohol use disorders in adolescence and later in adulthood, predisposing users to chronic diseases (79–81). Adolescents who drink heavily are at risk for identifiable health problems, with female adolescents at a greater risk of incurring more severe physical consequences (82). More disturbing is that alcohol use is often considered a gateway to the use of illegal substances because of the likelihood of deciding to use other drugs (83, 84). If alcohol intoxication continues in one's life, that can produce diminished inhibition, increased violent behavior, and poor judgment that can result in being in the wrong place at the wrong time, these factors all contribute to young deaths and injuries due to alcohol-related aggressive behavior (78).

Our study has several limitations. This study could only report on the inferences due to the use of a cross-sectional study design. The low number of schools used in this study constraints on generalizability and moreover that the study was limited to a group of black adolescents in township (i.e., peri-urban) setting. The current study only looked at proximal risk factors and not more distal risk factors, such as pricing, marketing, and availability. Information on alcohol use among the adolescents was self-reported, which may have been subject to recall bias. Response bias was likely because of the problematic nature of self-reported behavior, as some students might not have responded honestly. Social desirability as a bias that lead to underreporting or overreporting socially undesirable attributes might have affected the prevalence of alcohol use and other information. The use of a comprehensive tool that includes the quantitative measure, frequency, and types, to screen for alcohol use, such as the Alcohol Use Disorders Identification Test, should be considered in future prospective studies. There is a need for an analytical approach and rigorous application of standardized methods to assess alcohol use (85), among adolescents. We acknowledge the limitation of not testing the reliability of the questionnaire statistically, as one of the characteristics of a valid questionnaire (86), which was not done in the original studies (4, 16, 40), from where we adapted the questionnaire used in the current study. However, the correspondences of prevalence rates and risk factors with other studies provide support to the current findings.

## Conclusion

The current study reported high prevalence of alcohol use, binging, and cannabis use, especially among the male adolescents in the township high schools. The study further showed early use of alcohol, as well as an increased risk of continued drinking throughout the adolescence phase. In agreement with few studies conducted in the township high schools (16, 18, 27), adolescent drinking should be given a priority as one of the major public health concerns in South Africa. Risk factors for alcohol use among adolescents were sex, age, school grade, repeated grade, and working during spare time. These findings are suggestive of more evidence-based interventions that would have greater impact in addressing alcohol use among adolescents, such as focusing on availability, marketing, and taxation of alcoholic beverages.

## Data availability statement

The raw data supporting the conclusions of this article will be made available by the authors, without undue reservation.

## Ethics statement

The studies involving human participants were reviewed and approved by Sefako Makgatho Health Sciences University Research and Ethics Committee (SMUREC/H/51/2018: PG), and received permission from Tshwane Department of Education (Ref 8/4/4/1/2). Written informed consent to participate in this study was provided by the participants' legal guardian/next of kin.

## Author contributions

BM developed the proposal and undertook the data collection, data capturing, and project administration. MM initiated the research concept, supervised the study, and initiated the first draft of the manuscript. LC initiated the first draft of the manuscript and performed the initial data analysis. PM contributed to the first draft of the manuscript, performed the initial data analysis, validated data analysis, and reviewed and approved the final manuscript. All authors contributed to the article and approved the submitted version.

## Funding

This research was self-funded.

## Conflict of interest

The authors declare that the research was conducted in the absence of any commercial or financial relationships that could be construed as a potential conflict of interest. The handling editor KM declared a past co-authorship with the author(s) PM.

## Publisher's note

All claims expressed in this article are solely those of the authors and do not necessarily represent those of their affiliated organizations, or those of the publisher, the editors and the reviewers. Any product that may be evaluated in this article, or claim that may be made by its manufacturer, is not guaranteed or endorsed by the publisher.

## References

[1] NIAAA. Underage Drinking. National Institute of Alcohol Abuse and Alcoholism (2006). Available online at: https://pubsniaaanihgov/publications/AA67/AA67htm (accessed May 30, 2022).

[2] UN. UNODC World Drug Report 2020: Global drug use rising; while COVID-19 has far reaching impact on global drug markets. United Nations Office on Drugs and Crime (2020). Available online at: https://wwwunodcorg/unodc/press/releases/2020/June/media-advisory—global-launch-of-the-2020-world-drug-reporthtml (accessed May 30, 2022).

[3] Gore FM Bloem PJ Patton GC Ferguson J Joseph V Coffey C. Global burden of disease in young people aged 10–24 years: a systematic analysis. Lancet. (2011) 377:2093–102. 10.1016/S0140-6736(11)60512-6 21652063

[4] Chauke TM van der Heever H Hoque ME. Alcohol use amongst learners in rural high school in South Africa. African J Primary Health Care Family Med. (2015) 7:1–6. 10.4102/phcfm.v7i1.755 26466397 PMC4656918

[5] Morojele NK Ramsoomar L. Addressing adolescent alcohol use in South Africa. SAMJ: South African Med J. (2016) 106:551–3. 10.7196/SAMJ.2016.v106i6.10944

[6] Miller JW Naimi TS Brewer RD Jones SE. Binge drinking and associated health risk behaviors among high school students. Pediatrics. (2007) 119:76–85. 10.1542/peds.2006-1517 17200273

[7] UNICEF. Improving child nutrition: the achievable imperative for global progress. New York: UNICEF (2013). p. 114.

[8] Maserumule OM Skaal L Sithole SL. Alcohol use among high school learners in rural areas of Limpopo province. South African J Psychiatry. (2019) 25:1–6. 10.4102/sajpsychiatry.v25i0.1183 31745441 PMC6852002

[9] Reda AA Moges A Wondmagegn BY Biadgilign S. Alcohol drinking patterns among high school students in Ethiopia: a cross-sectional study. BMC Public Health. (2012) 12:213. 10.1186/1471-2458-12-213 22433230 PMC3328246

[10] Young C de Klerk V. Patterns of alcohol use on a South African university campus: the findings of two annual drinking surveys. African J Drug Alcohol Stud. (2008) 7:101–12. 10.4314/ajdas.v7i2.46367

[11] Peltzer K Ramlagan S. Alcohol use trends in South Africa. J Social Sci. (2009) 18:1–12. 10.1080/09718923.2009.11892661

[12] Ghuman S Meyer-Weitz A Knight S. Prevalence patterns and predictors of alcohol use and abuse among secondary school students in southern KwaZulu-Natal, South Africa: Demographic factors and the influence of parents and peers. South African Family Practice. (2014) 54:132–8. 10.1080/20786204.2012.10874192

[13] Onya H Tessera A Myers B Flisher A. Adolescent alcohol use in rural South African high schools: original. Afr J Psychiatry. (2012) 15:352–7. 10.4314/ajpsy.v15i5.44 23044890

[14] Reddy S James S Sewpaul R Sifunda S Ellahebokus A Kambaran NS. Umthente uhlaba usamila: the 3rd South African national youth risk behaviour survey 2011. (2013).

[15] Department Of Trade Industry. Final liquor policy paper national liquor policy review. (2016). Available online at: https://wwwgovza/sites/default/files/gcis_document/201609/40321gon1208pdf (accessed June 1, 2022).

[16] Mohale D Mokwena KE. Substance use amongst high school learners in the south of Johannesburg: Is this the new norm? South African family practice. Official J South African Acad Family Practice/Primary Care. (2020) 62:e1–6. 10.4102/safp.v62i1.5122 33314944 PMC8378201

[17] Obot IS Saxena S. Substance use among young people in urban environments. Substance Use Among Young People in Urban Environments: A Brief Survey in Nine, 1st ed. Geneva: World Health Organization. (2005). Available online at: countrieshttp://citeseerxistpsuedu/viewdoc/download?doi=10113784244&rep=rep1&type=pdf (accessed May 30, 2022).

[18] Ramsoomar L Morojele NK Norris SA. Alcohol use in early and late adolescence among the birth to twenty cohort in Soweto, South Africa. Global Health Action. (2013) 6:19274. 10.3402/gha.v6i0.19274 23364084 PMC3556711

[19] Balsa AI Giuliano LM French MT. The effects of alcohol use on academic achievement in high school. Econ Educ Rev. (2011) 30:1–15. 10.1016/j.econedurev.2010.06.015 21278841 PMC3026599

[20] Crosnoe R. The connection between academic failure and adolescent drinking in secondary school. Sociol Educ. (2006) 79:44–60. 10.1177/003804070607900103 20216913 PMC2834180

[21] Wegner L Flisher AJ Chikobvu P Lombard C King G. Leisure boredom and high school dropout in Cape Town, South Africa. J Adolesc. (2008) 31:421–31. 10.1016/j.adolescence.2007.09.004 18001827

[22] Pender NJ Murdaugh CL Parsons MA. Health Promotion in Nursing Practice. (2006).

[23] Arrioja Morales G Galera SAF Torres Reyes A. Gargantúa Aguila SDR, Avila Arroyo MLD, Morales Castillo FA. Risk factors for alcohol consumption in adolescents students. SMAD Revista eletrônica saúde mental álcool e drogas. (2017) 13:22–9. 10.11606/issn.1806-6976.v13i1p22-29

[24] Delgado AO Jiménez ÁP Gaviño FL Queija IS. Estilos educativos materno y paterno: evaluación y relación con el ejuste adolescente. Ann Psychol. (2007) 23:49–56. Available online at: https://revistas.um.es/analesps/article/view/23201/22481

[25] Room R Babor T Rehm J. Alcohol and public health. Lancet. (2005) 365:519–30. 10.1016/S0140-6736(05)17870-2 15705462

[26] Marshall EJ. Adolescent alcohol use: risks and consequences. Alcohol Alcoholism. (2014) 49:160–4. 10.1093/alcalc/agt180 24402246

[27] Mokwena K Sindane P. Cigarette smoking and alcohol intake among high school learners in Pretoria, South Africa. Psychol Edu J. (2020) 57:531–6. 10.17762/pae.v57i7.83

[28] Moodley SV Matjila MJ Moosa M. Epidemiology of substance use among secondary school learners in Atteridgeville, Gauteng. South African J Psychiatry. (2012) 18:2–7. 10.4102/sajpsychiatry.v18i1.320

[29] Debeila S Modjadji P Madiba S. High prevalence of overall overweight/obesity and abdominal obesity amongst adolescents: an emerging nutritional problem in rural high schools in Limpopo Province, South Africa. African J Primary Health Care Family Med. (2021) 13:e1–9. 10.4102/phcfm.v13i1.2596 34082550 PMC8182488

[30] Keats E Rappaport A Shah S Oh C Jain R Bhutta Z. The dietary intake and practices of adolescent girls in low-and middle-income countries: a systematic review. Nutrients. (2018) 10:1978. 10.3390/nu10121978 30558128 PMC6315365

[31] Hayman LL Worel JN. Healthy lifestyle behaviors: the importance of individual and population approaches. J Cardiovas Nursing. (2014) 29:477–8. 10.1097/JCN.0000000000000199 25290617

[32] Larson KE Nguyen AJ Solis MGO Humphreys A Bradshaw CP Johnson SL. systematic literature review of school climate in low and middle income countries. Int J Educ Res. (2020) 102:101606. 10.1016/j.ijer.2020.101606

[33] Tomczyk S Isensee B Hanewinkel R. Moderation, mediation—or even both? School climate and the association between peer and adolescent alcohol use. Addict Behav. (2015) 51:120–6. 10.1016/j.addbeh.2015.07.026 26255636

[34] McLeroy KR Bibeau D Steckler A Glanz K. An ecological perspective on health promotion programs. Health Educ Q. (1988) 15:351–77. 10.1177/109019818801500401 3068205

[35] Bronfenbrenner U. The Ecology of Human Development: Experiments by Nature and Design. Cambridge: Harvard University Press (1979).

[36] Pernegger L. Godehart S. Townships in the South African Geographic Landscape—Physical and Social Legacies and Challenges. Cape Town, South Africa: Training for Township Renewal Initiative (2007).

[37] Philip K. A History of Townships in South Africa. Economics of South African Townships: Special Focus on Diepsloot. p. 31–49. 10.1596/978-1-4648-0301-7_ch1

[38] Dass S Ringuest A. Basic Education Rights Handbook: Education rights in South Africa Johannesburg, South Africa: SECTION27 (2017). Available online at: http://section27orgza/wpcontent/uploads/2017/02 (accessed May 30, 2022).

[39] KFPP. Stages of Adolescence. Kids First Pediatric Partners. Available online at: https://wwwkidsfirstpediatricpartnerscom/parent-education/stages-adolescence/ (accessed May 30, 2022).

[40] Heever HVD. Alcohol use among students at the University of Limpopo, South Africa: substance abuse. African J Phys Health Edu Recreat Dance. (2014) 20:364–73. Available online at: https://hdl.handle.net/10520/EJC162263

[41] Perneger TV Courvoisier DS Hudelson PM Gayet-Ageron A. Sample size for pre-tests of questionnaires. Qual Life Res. (2015) 24:147–51. 10.1007/s11136-014-0752-2 25008261

[42] Modjadji P Mashishi J. Persistent malnutrition and associated factors among children under five years attending primary health care facilities in Limpopo Province, South Africa. Int J Environ Res Public Health. (2020) 17:7580. 10.3390/ijerph17207580 33086477 PMC7589291

[43] Bendel RB Afifi AA. Comparison of stopping rules in forward “stepwise” regression. J Am Stat Assoc. (1977) 72:46–53. 10.1080/01621459.1977.10479905

[44] Mickey RM Greenland S. The impact of confounder selection criteria on effect estimation. Am J Epidemiol. (1989) 129:125–37. 10.1093/oxfordjournals.aje.a115101 2910056

[45] Hosmer Jr DW Lemeshow S Sturdivant RX. Applied Logistic Regression. Hoboken: John Wiley & Sons (2013). 10.1002/9781118548387

[46] World Medical Association Declaration of Helsinki. Ethical Principles for Medical Research Involving Human Subjects. Available online at: http://wwwwmanet/e/policy/b3htm2008 (accessed March, 2022).

[47] Adebanke O-I Ogundipe O Amoo E Adeloye D. Substance use among adolescents in sub-Saharan Africa: a systematic review and meta-analysis. South African J Child Health. (2018) 12:79. 10.7196/SAJCH.2018.v12i2b.1524

[48] Hibell B Guttormsson U. The 2011 ESPAD Report: Substance use among students in 36 European Countries (ESPAD Report 2012).

[49] Ladikos A Prinsloo J Neser J Van der Merwe E Ovens M. Under-age drinking in schools: an exploratory survey. Acta Crim: Afr J Criminol Victimology. (2003) 16:123–35. Available online at: https://hdl.handle.net/10520/EJC28805

[50] Pengpid S Peltzer K. Alcohol use and associated factors among adolescent students in Thailand. West Indian Med J. (2012) 61:890–6. 10.7727/wimj.2012.059 24020229

[51] Hingson R Heeren T Levenson S Jamanka A Voas R. Age of drinking onset, driving after drinking, and involvement in alcohol related motor-vehicle crashes. Accident Anal Prevent. (2002) 34:85–92. 10.1016/S0001-4575(01)00002-1 11789578

[52] Hingson R Heeren T Winter MR Wechsler H. Early age of first drunkenness as a factor in college students' unplanned and unprotected sex attributable to drinking. Pediatrics. (2003) 111:34–41. 10.1542/peds.111.1.34 12509551

[53] Hingson R Heeren T Zakocs R. Age of drinking onset and involvement in physical fights after drinking. Pediatrics. (2001) 108:872–7. 10.1542/peds.108.4.872 11581438

[54] Mogotsi M Nel K Basson W Tebele C. Alcohol use by students at an emerging university in South Africa. J Sociol Social Anthropol. (2014) 5:187–95. 10.1080/09766634.2014.11885623

[55] Harker N Londani M Morojele N Petersen Williams P Parry CD. Characteristics and predictors of heavy episodic drinking (HED) among young people aged 16–25: the international alcohol control study (IAC), Tshwane, South Africa. Int J Environ Res Public Health. (2020) 17:3537. 10.3390/ijerph17103537 32438540 PMC7277734

[56] Morojele NK Lombard C Harker Burnhams N Petersen Williams P Nel E Parry CDH. Alcohol marketing and adolescent alcohol consumption: results from the international alcohol control study (South Africa). South African Med J. (2018) 108:782–8. 10.7196/SAMJ.2018.v108i9.12958 30182905

[57] Chung T Creswell KG Bachrach R Clark DB Martin CS. Adolescent binge drinking. Alcohol Res Current Rev. (2018) 39:5–15. 10.35946/arcr.v39.1.02 PMC6104966 30557142

[58] Hingson R White A. New research findings since the 2007 surgeon general's call to action to prevent and reduce underage drinking: a review. J Stud Alcohol Drugs. (2014) 75:158–69. 10.15288/jsad.2014.75.158 24411808 PMC3893630

[59] Siqueira L Smith VC Levy S Ammerman SD Gonzalez PK Ryan SA. Binge drinking. Pediatrics. (2015) 136:e718–e26. 10.1542/peds.2015-2337 26324872

[60] Organization WH. Global Strategy to Reduce the Harmful Use of Alcohol. Geneva: World Health Organization (2010). 10.2471/BLT.19.241737 PMC7047030 32132758

[61] Flisher AJ Parry CD Evans J Muller M Lombard C. Substance use by adolescents in Cape Town: prevalence and correlates. J Adolesc Health. (2003) 32:58–65. 10.1016/S1054-139X(02)00445-7 12507802

[62] Morojele N Parry C Brook J. Substance abuse and the young: taking action. MRC Res Brief. (2009) 6:1–4.

[63] Dida N Kassa Y Sirak T Zerga E Dessalegn T. Substance use and associated factors among preparatory school students in Bale Zone, Oromia Regional State, Southeast Ethiopia. Harm Reduct J. (2014) 11:1–6. 10.1186/1477-7517-11-21 25108629 PMC4131802

[64] Osei-Bonsu E Appiah PK Norman ID Asalu GA Kweku M Ahiabor SY. Prevalence of alcohol consumption and factors influencing alcohol use among the youth in Tokorni-Hohoe, Volta region of Ghana. Sci J Public Health. (2017) 5:205–14. 10.11648/j.sjph.20170503.18

[65] Manu E Maluleke XT Douglas M. Knowledge of high school learners regarding substance use within high school premises in the Buffalo Flats of East London, Eastern Cape Province, South Africa. J Child Adolesc Subst Abuse. (2017) 26:1–10. 10.1080/1067828X.2016.1175984

[66] Olumide AO Robinson AC Levy PA Mashimbye L Brahmbhatt H Lian Q. Predictors of substance use among vulnerable adolescents in five cities: findings from the well-being of adolescents in vulnerable environments study. J Adolescent Health. (2014) 55:S39–47. 10.1016/j.jadohealth.2014.08.024 25454001 PMC4493747

[67] Mothibi K. Substance abuse amongst high school learners in rural communities. Universal J Psychol. (2014) 2:181–91. 10.13189/ujp.2014.020601

[68] Jacobus J Bava S Cohen-Zion M Mahmood O Tapert SF. Functional consequences of marijuana use in adolescents. Pharmacol Biochem Behav. (2009) 92:559–65. 10.1016/j.pbb.2009.04.001 19348837 PMC2697065

[69] YRBS. Making the Connection: Alcohol Behaviors and Academic Grades. (2019). Available online at: https://wwwcdcgov/healthyschools/health_and_academics/pdf/320889-D_FS_Alcohol_Behaviors_508tagpdf (accessed May 30, 2022).

[70] El Ansari W Stock C Mills C. Is alcohol consumption associated with poor academic achievement in university students? Int J Prev Med. (2013) 4:1175–88. 24319558 PMC3843305

[71] Paternoster R Bushway S Brame R Apel R. The effect of teenage employment on delinquency and problem behaviors. Social Forces. (2003) 82:297–335. 10.1353/sof.2003.010423825897

[72] Donath C Gräßel E Baier D Pfeiffer C Bleich S Hillemacher T. Predictors of binge drinking in adolescents: ultimate and distal factors—a representative study. BMC Public Health. (2012) 12:263. 10.1186/1471-2458-12-263 22469235 PMC3378431

[73] Hemphill SA Heerde JA Scholes-Balog KE Herrenkohl TI Toumbourou JW. Catalano Jr. RF. Effects of early adolescent alcohol use on mid-adolescent school performance and connection: a longitudinal study of students in Victoria, Australia and Washington State, United States. J School Health. (2014) 84:706–15. 10.1111/josh.12201 25274170 PMC4196706

[74] Holtes M Bannink R. Joosten - van Zwanenburg E, van As E, Raat H, Broeren S. Associations of truancy, perceived school performance, and mental health with alcohol consumption among adolescents. J School Health. (2015) 85:852–60. 10.1111/josh.12341 26522174

[75] Wolaver AM. Effects of heavy drinking in college on study effort, grade point average, and major choice. Contemp Econ Policy. (2002) 20:415–28. 10.1093/cep/20.4.415

[76] Brown SA Tapert SF Granholm E Delis DC. Neurocognitive functioning of adolescents: effects of protracted alcohol use. Alcoholism Clin Exp Res. (2000) 24:164–71. 10.1111/j.1530-0277.2000.tb04586.x 10698367

[77] Patton GC Sawyer SM Santelli JS Ross DA Afifi R Allen NB. Our future: a Lancet commission on adolescent health and wellbeing. Lancet. (2016) 387:2423–78. 10.1016/S0140-6736(16)00579-1 27174304 PMC5832967

[78] Council US Institute of Medicine Committee. U. Health Consequences of Adolescent Alcohol Involvement. (2004). Available online at: https://wwwncbinlmnihgov/books/NBK37610/ (accessed May 2022).

[79] Martins JG Guimarães MO Jorge KO Silva CJdP Ferreira RC Pordeus IA. Binge drinking, alcohol outlet density and associated factors: a multilevel analysis among adolescents in Belo Horizonte, Minas Gerais State, Brazil. Cadernos de Saúde Pública. (2019) 36:e00052119. 10.1590/0102-311x00052119 31939545

[80] Newbury-Birch D. Impact of Alcohol Consumption on Young People: A Review of Reviews. (2009).

[81] Rehm J. How should prevalence of alcohol use disorders be assessed globally? Int J Methods Psychiatr Res. (2016) 25:79–85. 10.1002/mpr.1508 27133364 PMC6877138

[82] Aarons GA Brown SA Coe MT Myers MG Garland AF Ezzet-Lofstram R. Adolescent alcohol and drug abuse and health. J Adolesc Health. (1999) 24:412–21. 10.1016/S1054-139X(99)00006-3 10401969

[83] Kandel DB Davies M. From adolescence to adulthood. Am J Psychiatry. (1996) 153:1654. 10.1176/ajp.153.12.1654a 8942471

[84] Brown SA Tapert SF Tate SR Abrantes AM. The role of alcohol in adolescent relapse and outcome. J Psychoactive Drugs. (2000) 32:107–15. 10.1080/02791072.2000.10400216 10801072

[85] Modjadji P Pitso M. Maternal tobacco and alcohol use in relation to child malnutrition in Gauteng, South Africa: a retrospective analysis. Children. (2021) 8:133. 10.3390/children8020133 33670265 PMC7918556

[86] de Yébenes Prous MJ Salvanés FR Ortells LC. Validation of questionnaires. Reumatol Clí*nica*. (2009) 5:171–7. 10.1016/j.reuma.2008.09.007 21794604

